# A combined morphological and molecular approach for hair identification to comply with the European ban on dog and cat fur trade

**DOI:** 10.7717/peerj.7955

**Published:** 2019-11-11

**Authors:** Alessia Mariacher, Luisa Garofalo, Rita Fanelli, Rita Lorenzini, Rosario Fico

**Affiliations:** 1 Centro di Referenza Nazionale per la Medicina Forense Veterinaria, Istituto Zooprofilattico Sperimentale delle Regioni Lazio e Toscana, Grosseto, Italy; 2 Centro di Referenza Nazionale per la Medicina Forense Veterinaria, Istituto Zooprofilattico Sperimentale delle Regioni Lazio e Toscana, Rieti, Italy

**Keywords:** *Canis lupus*, *Felis silvestris*, Genetic analysis, Hair morphology, Illegal trade, Microscopy, Veterinary forensics

## Abstract

Animal furs are encountering more and more the detriment of public opinion, that is increasingly sensitive to animals, their welfare and protection. The feeling of outrage against animal suffering is particularly intense when cats and dogs are involved, since these are the most popular pets in Western countries. However, in some Asian countries breeding of dogs and cats for the fur industry is a common practice. These furs and their finished garments are often mislabelled in order to be imported and sold to unaware consumers in Western countries. The European Union has issued the Regulation 1523/2007, which bans the use and trade of dog and cat furs. The main purposes of the Regulation were to normalise the internal market and to address the concerns of European consumers about the risk of inadvertently buying products containing these species. The Regulation states that several analytical methods (microscopy, DNA testing and mass spectrometry) can be used to exclude dogs and cats as source species, but an official analytical protocol was not provided. In this paper, we report on the development of a reliable and affordable method for species identification in furs, based on a combined morphological and molecular approach. Our protocol provides an initial morphological analysis as a time and cost effective screening test. Only samples that are morphologically not excluded as canid/felid furs, based on few selected microscopic features, are then submitted to DNA testing. The application of this protocol on seized furs reached 92% identification of species. Our approach assists in identifying frauds and reinforcing the ban on dog and cat fur trade, allowing (1) rapid inexpensive recognition of fake furs, (2) exclusion of non-canid/non-felid furs through fast microscopic morphological screening, (3) overall cost reduction with lower number of samples to be submitted to DNA analysis, (4) analytical protocol to stand in court in case criminal sanctions are to be applied.

## Introduction

The fur market is a flourishing activity in several Asian countries, and the breeding of dogs and cats for the production of furs is legal and widely practised. Currently, China is the largest producer and exporter to Western countries of fur items, including soft-toys and clothing accessories (People for the Ethical Treatment of Animals (PETA), 2019). In Europe, on the contrary, dogs and cats are popular and treasured family pets, with 23−25% households owning at least one cat or one dog, respectively (Fediaf, 2018). Therefore, consumers demand to be assured about the production of furs, in order to avoid the risk of inadvertently buying products made with fur from these animals (European Parliament, 2007).

To counteract the growing concern of consumers, European Union (EU) officially banned the import and export from all Member States of dog (*Canis lupus familiaris*) and cat (*Felis silvestris*) furs, and all products containing fur from these species, with the Regulation 1523/2007 (European Parliament, 2007), applying since 31st December 2008. The Regulation was also intended to harmonize the measures to prohibit cat and dog furs at a European level, since several EU Member States had adopted different national legislations on the matter. EU Member States were left free to define their own penalties for infringements, and these can range from administrative sanctions up to 20,000 Euros to criminal sanctions including fines or up to 3 years imprisonment (European Commission, 2013).

The EU Regulation suggests that one or more analytical methods can be used to identify dog and cat furs, amongst microscopy (morphological analysis of animal hairs), molecular testing (PCR-based DNA analysis) and MALDI-TOF mass spectrometry (quantitative analysis of hair keratin peptides, Hollemeyer, Altmeyerb & Heinzle, 2007). EU Member States can adopt one or more techniques, yet information on the methods actually applied or on their effectiveness is scarce. A Report from the European Commission (2013) states that the number of analyses carried out on imported furs in the first years after the Regulation entered in force was very low. For example, in Italy only 20 samples were analysed in the period 2009−2010, but the indication of the type of analyses carried out and their outcomes is not available. The low number of analyses on imported furs contrasts with the large numbers reported by the press or animal welfare associations relating to Chinese export of fur products derived from dogs and cats. The Report also recognises that the number of official controls on the placing on the EU market of dog and cat furs has been inadequate, in particular as regards internet sales. Unfortunately, EU Member States did not provide specific data on the numbers of dog and cat furs identified during controls. The only fact that can be extrapolated from the Report to this effect is that in France in 2009 out of 46 analysed samples, 17 contained dog or cat hair, that is, 37% of the samples analysed (European Commission, 2013).

The availability of verified analytical methods to discriminate dog and cat furs from allowed fur-bearing species is an essential step to comply with the ban. This is not an easy task, since some of the licit animals used by the fur industry are phenotypically similar and/or phylogenetically closely related to the dog, like other species belonging to canid or felid families. Furthermore, furs and pelts (especially when used in small items such as coat collars, scarves, key-rings or children’s toys) may have been treated with aggressive chemicals, dyed, trimmed or otherwise altered, thus making the identification of species tough, whatever the method used.

In order to comply with the EU Regulation, we explored a combined morphological and molecular approach for species identification in furs. Our approach is designed to be applied in its first step (morphological analysis) by a surveillance network spread throughout the territory. Namely, morphological analysis of fur samples could be carried out in any laboratory equipped with basic logistic and technical facilities. Morphology would serve as a time- and cost-effective screening test to rule out some of the legally employed fur-bearing species through the microscopic examination of few morphological characteristics of the hair structure, which could be observed even by minimally trained personnel. Samples that could not be morphologically excluded as canid or felid furs based on selected characteristics would be then submitted to a molecular-equipped laboratory for DNA testing.

In non-human DNA forensics, genetic identification of species is required when biological evidence samples are too small or degraded and do not allow recognition of morphological distinctive features, like in tanned fur products (Linacre & Tobe, 2011). In these cases, species identification is frequently achieved by amplification and sequencing of short but informative DNA fragments, either coding or neutral (see Iyengar, 2014; Johnson, Wilson-Wilde & Linacre, 2014 for reviews). Mitochondrial markers are widely used for the identification of species in animal forensics (Wilson et al., 1995; Johnson, Wilson-Wilde & Linacre, 2014) because of many advantages in their analysis (Alacs et al., 2010), including the possibility to compare results from unknown casework samples with reference sequences registered in public online databases (e.g., GenBank; http://www.ncbi.nlm.nih.gov/genbank/index.html).

The purpose of this article is to illustrate the procedure for morphological pre-selection of hairs to be submitted to further DNA testing when examining fur samples, to discern dog and cat furs from allowed fur-bearing species. The effective application of our combined morphological and molecular protocol on real casework samples is also depicted.

## Materials and Methods

Reference hairs from different species were obtained from dead or live animals of known species, identified by expert biologists or veterinarians. Dead animals were submitted to the Centro di Referenza Nazionale per la Medicina Forense Veterinaria (http://www.izslt.it/medicinaforense/) for forensic purposes. Whenever possible, hairs were taken from different areas of the body (such as back, abdomen and tail regions) and from more individuals belonging to the same species. Overall, hairs from 87 individuals belonging to 25 known (sub) species were used to build and test our morphological criterion.

Casework fur samples (*N* = 25) came from pelts or fur scraps withdrawn at customs by Convention on the International Trade in Endangered Species of Wild Fauna and Flora (CITES) agents, or seized by police from retailers.

Guard hairs from both reference and questioned (casework) samples were examined using a light microscope (Leica DM4000 B LED) at ×200 and ×400 magnification. According to Teerink (1991), guard hairs were identified as the long and stiff hair types in the sample, regardless of the sub-type of guard hairs (e.g., GH0, GH1 or GH2). The shield was identified as a thickening in the distal part of the hair, that is, toward the tip, and the shaft was termed as the thinner proximal part of the hair, i.e. toward the base (Teerink, 1991). Three major characteristics of guard hairs were observed in our morphological analysis: the cuticle pattern, the medullar pattern along all the length of the shaft, and the medullar margins in the thickest part of the shield region.

Two types of preparations have been set up: cuticle scale casts (to observe cuticle patterns), and whole mount specimens (to observe medullar patterns and medullar margins). The cuticle scale cast was obtained using a transparent water-based liquid glue (FluidFix; Alpa Collanti, Milano, Italy) as the printing medium. A fine coat of glue was spread onto an object slide and the hair was placed on the slide. Once the glue had hardened (in approximately 60 min), the hair was removed with tweezers, leaving its cast on the film. The whole mount specimens were obtained by mounting a hair on an object slide with a drop of xylene balm (Eukitt quick-hardening mounting medium, Sigma–Aldrich) and a drop of diluent (Clearene; Leica Biosystems, Wetzlar, Germany) added directly onto the slide, to avoid subsequent air cracks formation.

The terminology used to describe cuticle scale patterns and medullar characteristics was adopted from Moore, Spence & Dugnolle (1974) and Teerink (1991).

The 25 casework samples were examined in parallel with both the morphological method and DNA analysis, to test the two approaches, with the ultimate goal of combining them in a diagnostic procedure aimed at discriminating dog and cat furs from allowed fur-bearing species. We followed a molecular method (Garofalo et al., 2018) aimed at targeting canid and felid mitochondrial DNA with specific primer pairs, that deliberately avoid binding to human DNA. Human contamination, coming from handling of furs during production and supply chain, can complicate the interpretation of results. In brief, we applied a two-step DNA analysis that involved initially a nonspecific primer pair to identify the species through sequencing, then species-specific primer pairs to use in singleplex end-point PCRs for species confirmation as described by Garofalo et al. (2018).

Ethical clearance for this study was received from the “Istituto Zooprofilattico Sperimentale delle Regioni Lazio e Toscana”. All procedures for the handling of specimens and samples followed the criteria laid out by this Institute and the Italian Ministry of Health.

## Results

### Criteria chosen for morphological exclusion of dog and cat samples

**Figure 1:**
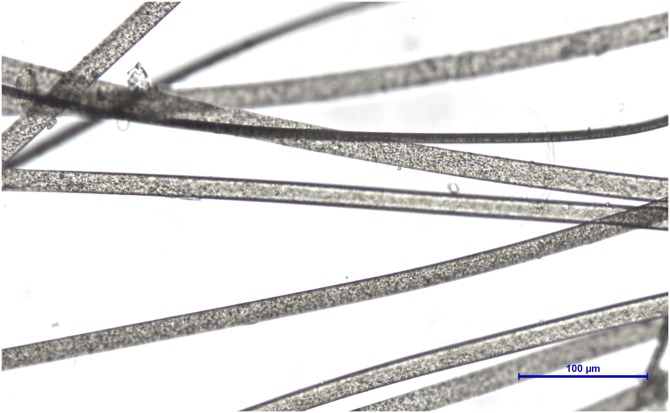
Synthetic fibres from fake fur (Photo credit, Alessia Mariacher). Light microscopy, whole mount, 40×.

**Figure 2:**
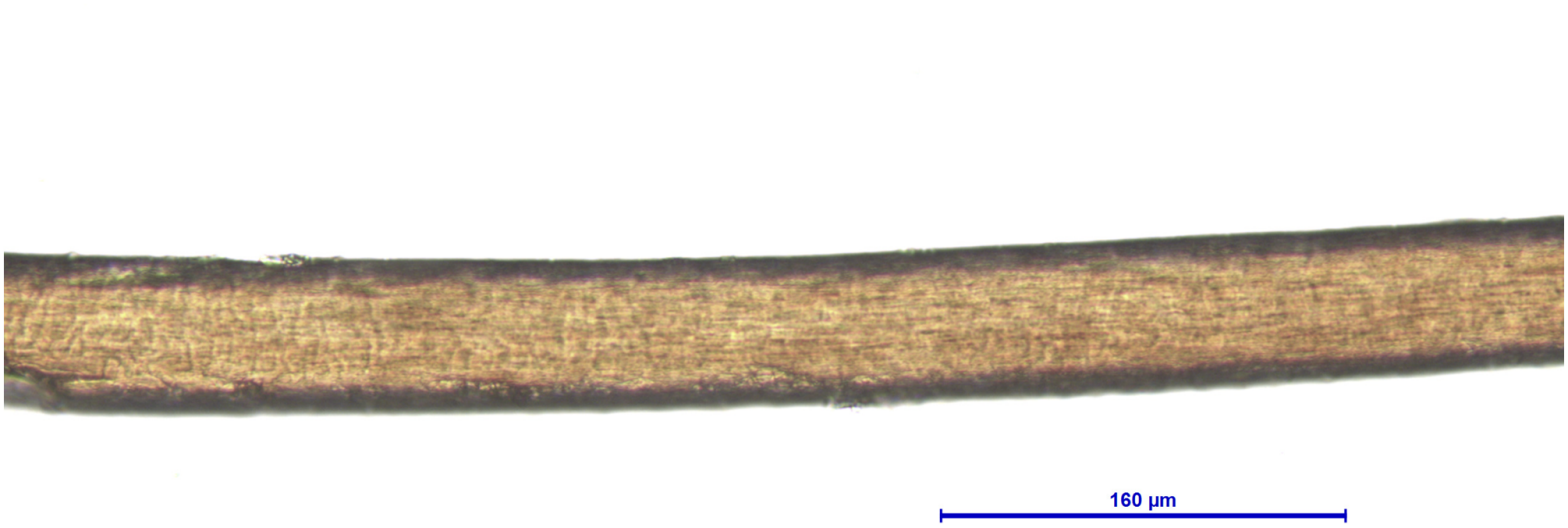
*Bos taurus*, guard hair (Photo credit, Alessia Mariacher). Light microscopy, whole mount, 40×.

**Figure 3:**
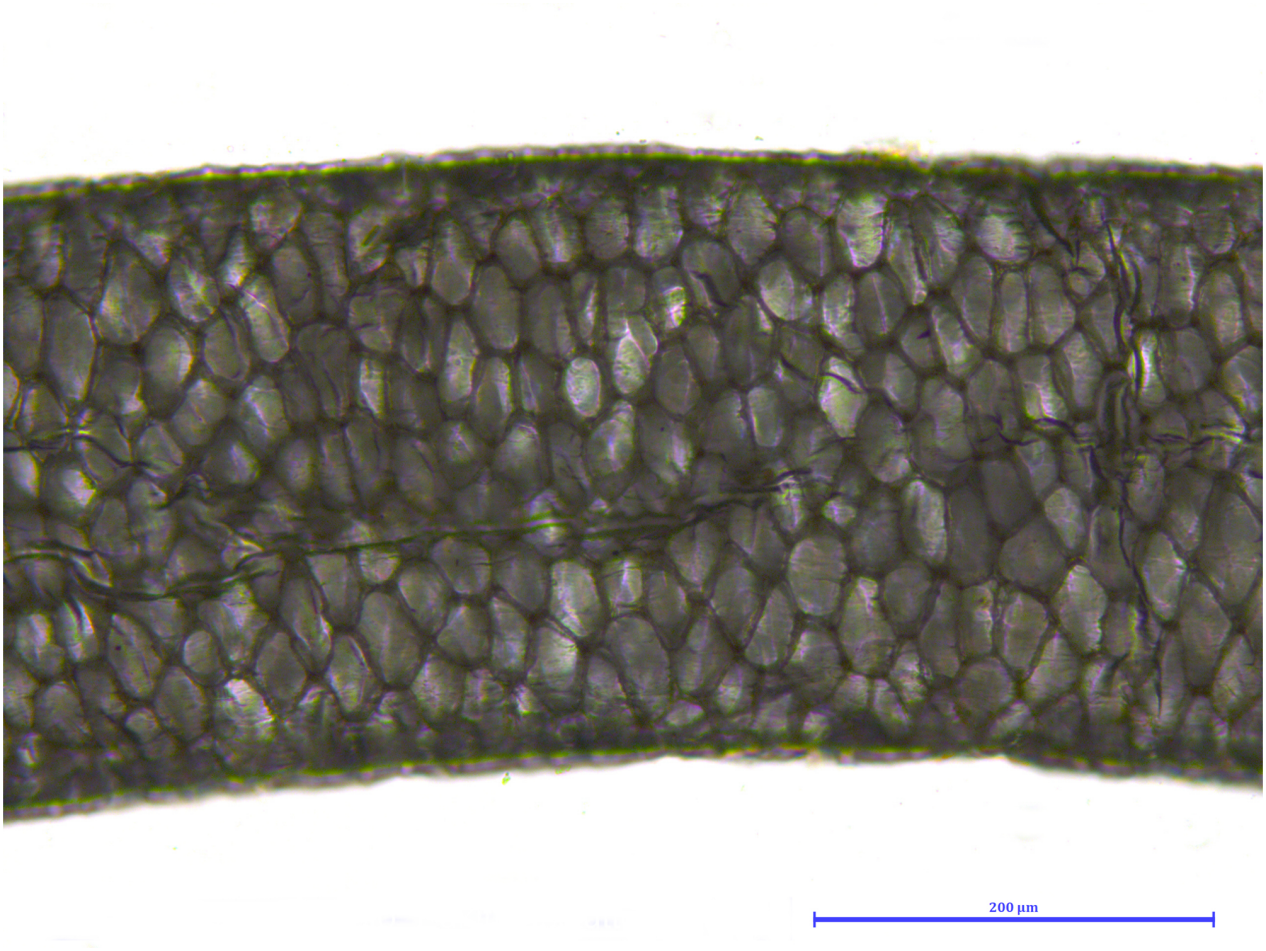
*Capreolus capreolus*, guard hair (Photo credit, Alessia Mariacher). Light microscopy, whole mount, 40×.

**Figure 4:**
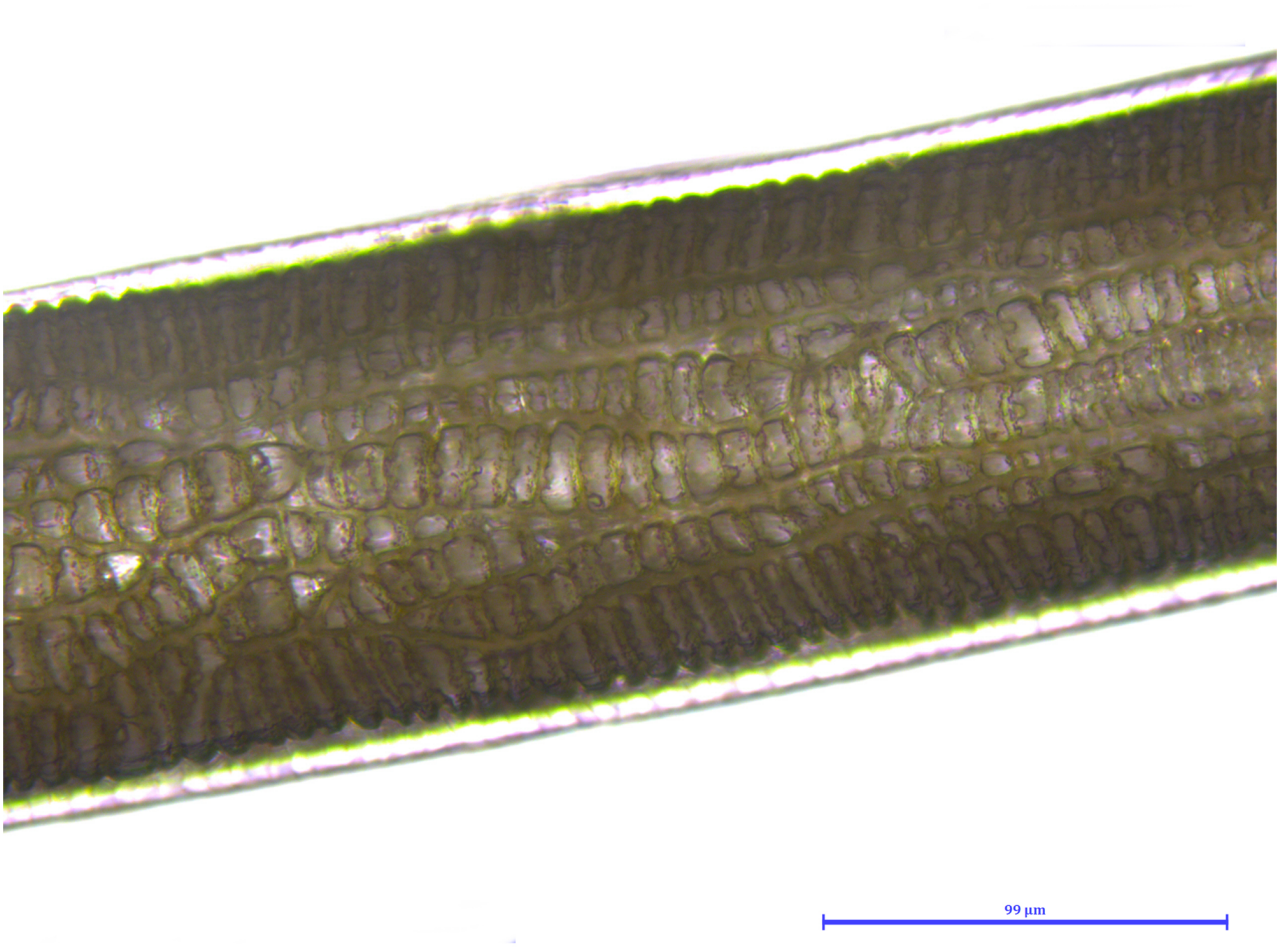
*Oryctolagus cuniculus*, guard hair, shield (Photo credit, Alessia Mariacher). Light microscopy, whole mount, 40×.

**Figure 5:**
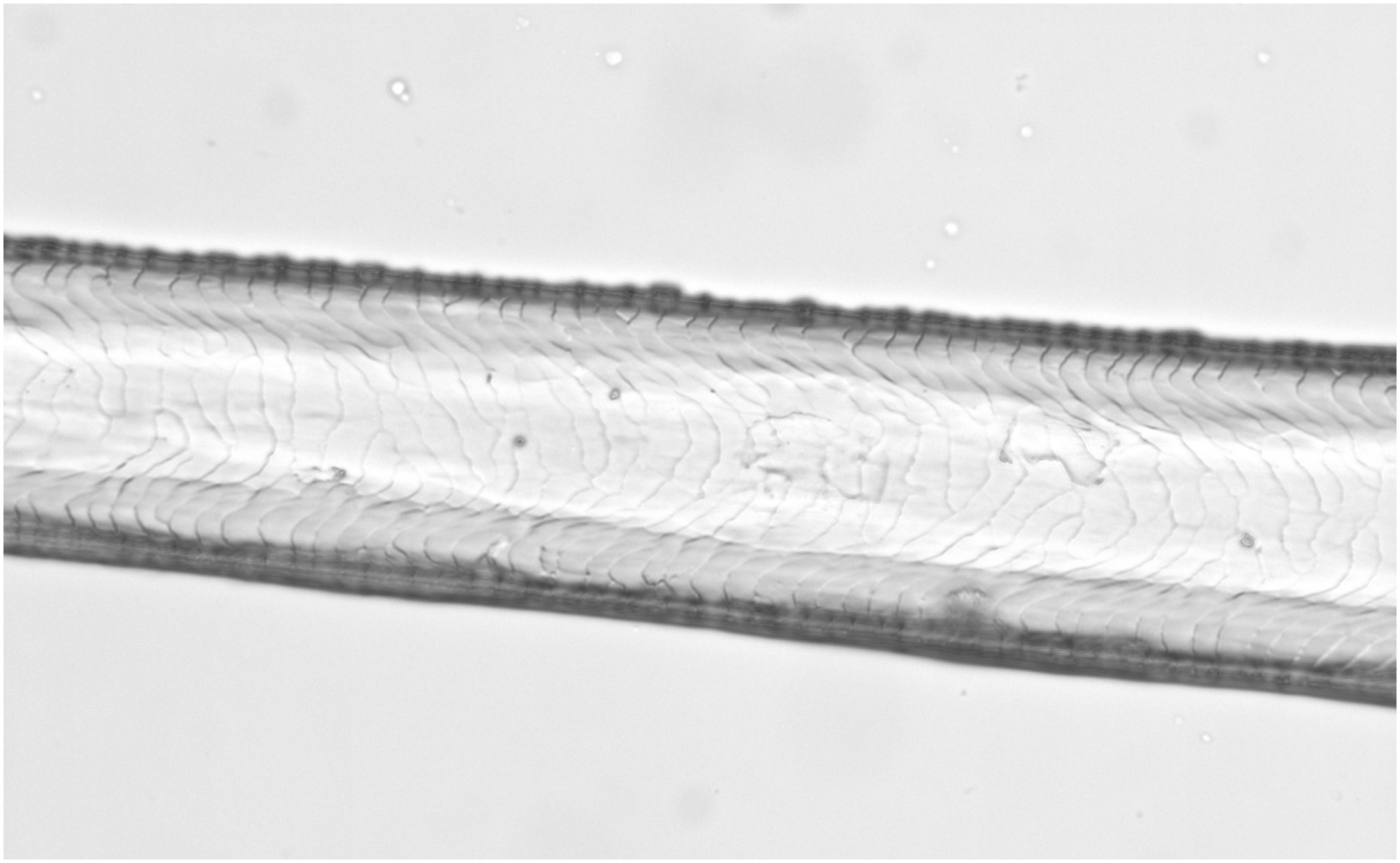
*Lepus europaeus*, guard hair, shield (Photo credit, Alessia Mariacher). Light microscopy, cuticle scale cast, 40×. Scale bar is not included in cuticular cast images, since the shaft may not be completely in contact with the printing medium and measures would be unreliable (Tridico et al., 2014).

**Figure 6:**
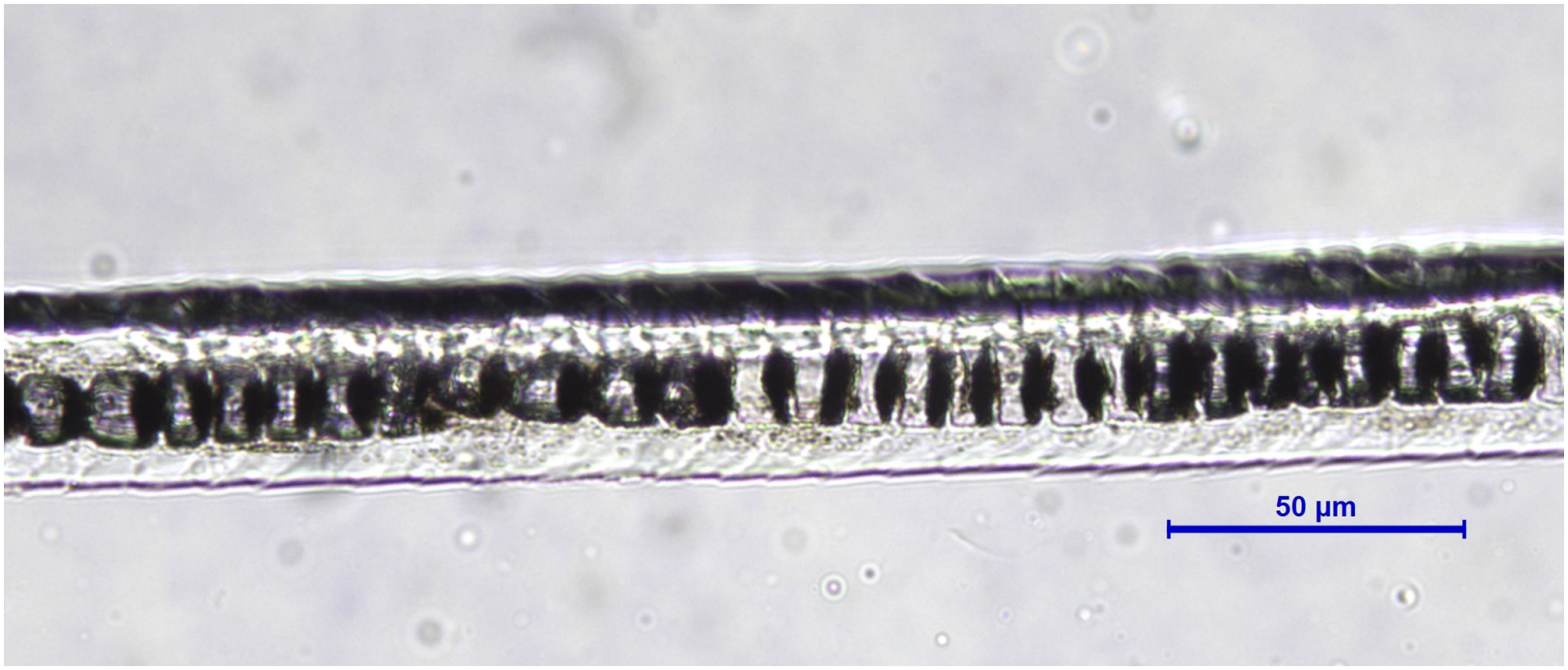
*Glis glis*, guard hair, shaft (Photo credit, Alessia Mariacher). Light microscopy, whole mount, 40×.

**Figure 7:**
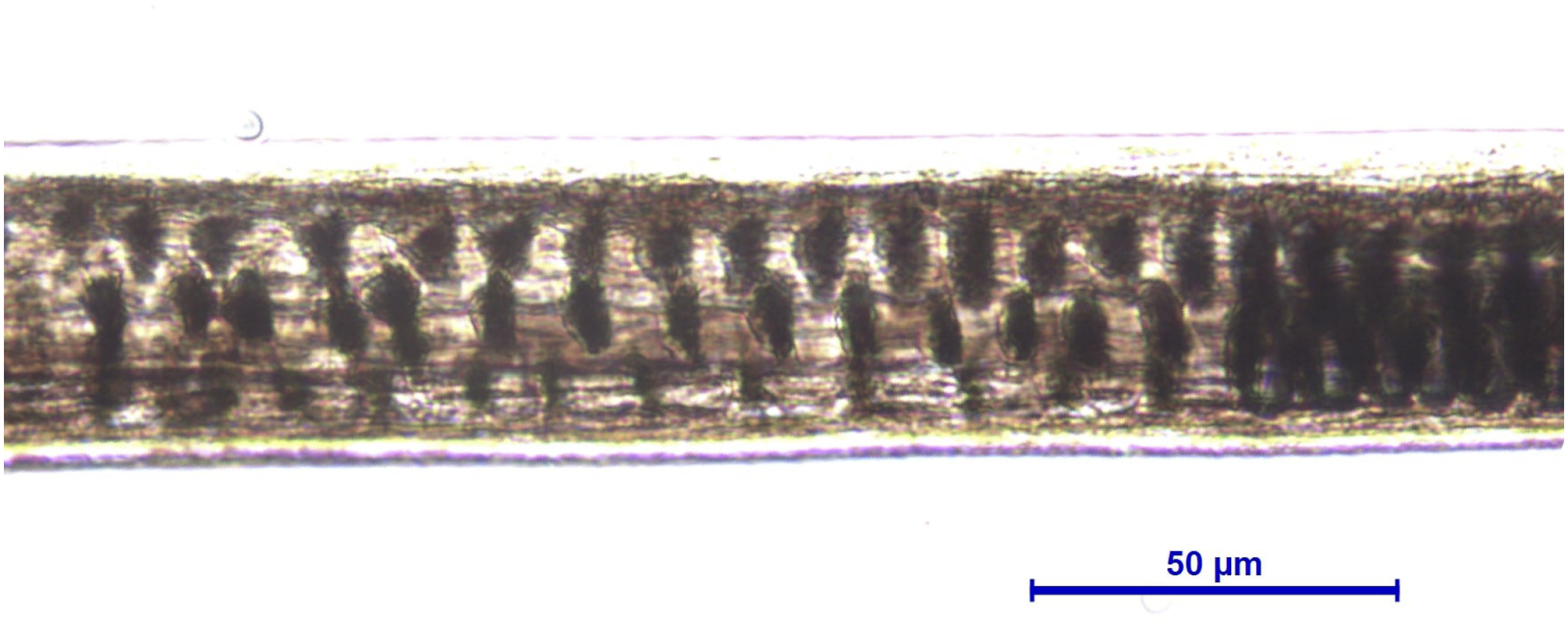
*Microtus arvalis*, guard hair, shield (Photo credit, Alessia Mariacher). Light microscopy, whole mount, 40×.

**Figure 8:**
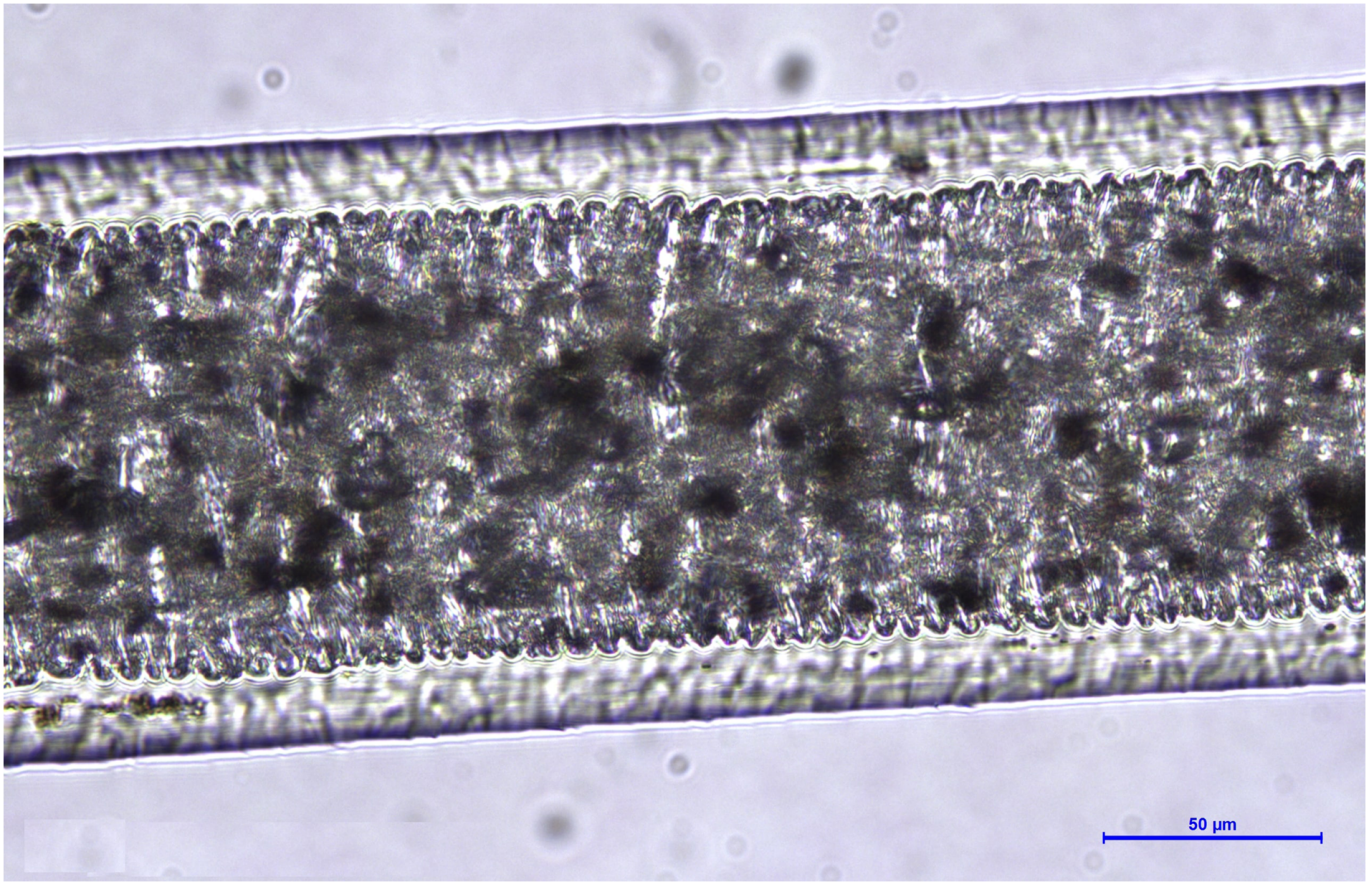
*Martes foina*, guard hair, shield (Photo credit, Alessia Mariacher). Light microscopy, whole mount, 20×.

**Figure 9:**
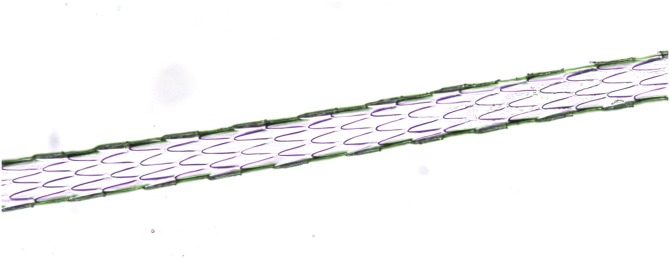
*Martes foina*, guard hair, shaft (Photo credit, Alessia Mariacher). Light microscopy, cuticle scale cast, 20×. Scale bar is not included in cuticular cast images, since the shaft may not be completely in contact with the printing medium and measures would be unreliable (Tridico et al., 2014).

Based on reference atlases and identification keys (Moore, 1988; Teerink, 1991; De Marinis & Agnelli, 1993; Van den Broeck, Mortier & Simoens, 2000; Toth, 2002; De Marinis & Asprea, 2006; Gonzàlez-Esteban, Villate & Irizar, 2006), we selected six cuticle/medullary arrangements that are usually not attributed to canid and felid hairs (Table 1, Figs. 1–9). It was considered in particular that the occurrence of the following morphological characteristics of guard hairs reasonably ensures the exclusion of dog and cat as source species of a fur sample: (1) absence of characteristic three layered internal structure of animal hair (lack of cuticle, cortex and medulla); (2) medulla absent for most of the hair length, (3) medulla filled in the entire hair, (4) medulla multicellular in regular rows with a longitudinal groove discernible on the shield, (5) medulla in shield isolated or crescent-shaped and (6) medullar margins strongly scalloped in the thickest part of the shield along with narrow diamond petal cuticular pattern in the hair shaft.

**Table 1:** Morphological criterion for exclusion of canid/felid hair. Morphological criterion based on six hair traits to classify fur samples that belong (NE = canid/felid not excluded) or do not belong (E = canid/felid excluded) to Canidae or Felidae. See text for details.

Cuticle and medulla features	Categorisation
1a. Fibres without characteristic internal structure (i.e. cuticle, cortex and medulla) (Fig. 1)	Fake fur
1b. Hairs with characteristic three layers structure (cuticle, cortex and medulla)	NE
2a. Medulla absent for most of the hair length (Fig. 2)	E
2b. Medulla present for most of the hair length	NE
3a. Medulla filled in entire hair (Fig. 3)	E
3b. Medulla otherwise	NE
4a. Medulla multicellular in regular rows (Fig. 4). A longitudinal groove can be appreciated on the shield in cuticle scale casts (Fig. 5)	E
4b. Medulla otherwise	NE
5a. Medulla uniserial or 2 cells-wide ladder in the shaft (Fig. 6), and isolated or crescent shaped in the shield (Fig. 7)	E
5b. Medulla otherwise	NE
6a. Medullar margins strongly scalloped in the thickest part of the shield (Fig. 8), and cuticular pattern narrow diamond petal in the shaft (Fig. 9)	E
6b. Medullar margins in the shield and cuticular pattern in the shaft otherwise	NE (submit to DNA analysis)

According to the chosen criterion, fibres devoid of any internal structure were discarded as fake furs at the very first step of morphological analysis (category 1a, Table 1). Samples that showed the other above described patterns (all categories “a” of Table 1) were excluded as putative canid/felid furs. Samples showing none of such patterns (categories “b”, Table 1) were collectively categorised as “not excluded”, thus indicating that dogs or cats might be the source of the fur and molecular tests are required to proceed with identification at the species level.

Our selective criterion was tested on reference guard hairs from 87 individuals belonging to 25 known species or subspecies (Table 2), most of them traditionally used in furriery. For each individual, two hairs were used for the cuticle scale cast and two hairs for the whole mount.

**Table 2:** Results of the application of our morphological criterion on reference hair samples. Reference hair samples tested with our morphological criterion. Four hairs were analysed for each sample. E = sample excluded as canid/felid hair (categories “a” of Table 1); NE = sample not excluded as canid/felid hair (categories “b” of Table 1).

Family	Species	Number of samples	Result	
			E	NE
Bovidae	*Bos taurus* (cow) *Capra hircus* (goat) *Ovis aries* (sheep) *Rupicapra pyrenaica* (chamois)	3 3 2 4	3 2 2 4	0 1 0 0
Camelidae	*Vicugna pacos* (alpaca)	3	0	3
Canidae	*Canis lupus* (wolf) *Canis lupus familiaris* (dog) *Vulpes vulpes* (red fox)	8 10 6	0 0 0	8 10 6
Cervidae	*Capreolus capreolus* (roe deer) *Cervus elaphus* (red deer)	3 6	3 6	0 0
Cricetidae	*Microtus arvalis* (common vole)	1	1	0
Equidae	*Equus caballus* (horse)	4	3	1
Felidae	*Felis silvestris catus* (domestic cat) *Panthera tigris* (tiger)	10 1	0 0	10 1
Gliridae	*Glis glis* (dormouse)	2	2	0
Leporidae	*Lepus europaeus* (brown hare) *Oryctolagus cuniculus* (rabbit)	3 1	3 1	0 0
Mustelidae	*Martes foina* (beech marten) *Mustela nivalis* (weasel) *Meles meles* (European badger)	1 3 5	1 3 0	0 0 5
Procyonidae	*Procyon lotor* (raccoon)	1	0	1
Sciuridae	*Sciurus vulgaris* (red squirrel)	1	1	0
Suidae	*Sus scrofa* (wild boar)	1	1	0
Talpidae	*Talpa europaea* (mole)	1	1	0
Ursidae	*Ursus arctos* (brown bear)	4	4	0
Total		87	41	46

Of the examined reference hairs, 15 species (*Bos taurus*, *Capreolus capreolus*, *Cervus elaphus*, *Glis glis*, *Lepus europaeus, Martes foina*, *Microtus arvalis*, *Mustela nivalis*, *Oryctolagus cuniculus*, *Ovis aries*, *Rupicapra pyrenaica*, *Sciurus vulgaris*, *Sus scrofa*, *Talpa europaea*, and *Ursus arctos*) were correctly excluded as canids or felids hairs. All canids and felids samples (*Canis lupus* ssp., *Vulpes vulpes*, *Felis silvestris* ssp., *Panthera tigris*) were correctly assigned to the category “not excluded”. All the samples from three non-canid/non-felid species (*Meles meles*, *Procyon lotor*, *Vicugna pacos*) did not show any of the medulla and cuticle traits selected in our criterion, so that they were necessarily classified as “not excluded”. For the same reason, one out of three examined samples of *Capra hircus* and one out of four examined samples of *Equus caballus* were incorrectly classified as “not excluded” as canids or felids.

In conclusion, we obtained 12.6% (11 out of 87) of false positives in the “not excluded” group, that is the number of species that our selective criterion failed to recognise as not belonging to canids or felids. On the contrary, no false negatives were recorded (i.e., canid or felid samples incorrectly excluded from subsequent DNA testing).

### Combined morphological and molecular analysis of questioned samples

Twenty-five casework samples were screened by microscopy according to our morphological criterion (Table 3). Six furs were artificially dyed and five dated before 1980; the species of origin was only declared for 10 items (Table 3).

**Table 3:** Results from casework samples tested with our combined protocol.

ID	Approximative age	Dyed	Declared as	Morphological categorisation	DNA result
FUR-1	1980−2016	no	Raccoon dog	NE	*Nyctereutes procyonoides*(raccoon dog)
FUR-2	1980−2016	no	Raccoon dog	NE	*Nyctereutes procyonoides*(raccoon dog)
FUR-3	1980−2016	no	Raccoon dog	NE	No amplification
FUR-4	1980−2016	no	nd	NE	*Nyctereutes procyonoides*(raccoon dog)
FUR-5	1980−2016	no	nd	NE	No amplification
FUR-6	1980–2016	no	nd	NE	*Canis lupus* ssp. (dog/wolf)
FUR-7	1980–2016	yes	nd	NE	*Canis lupus* ssp. (dog/wolf)
FUR-8	1980-2016	no	Coyote	NE	*Canis latrans* (coyote)
FUR-9	1980-2016	no	Squirrel	E	*Sciurus* spp. (squirrel)
FUR-10	1930-1980	no	Fox	NE	*Vulpes vulpes* (red fox)
FUR-11	1980–2016	no	Silver fox	NE	*Vulpes vulpes* (red fox)
FUR-12	1980–2016	yes	nd	NE*	*Alopex lagopus* (Arctic fox)
FUR-13	1980–2016	no	nd	NE	*Vulpes vulpes* (red fox)
FUR-14	1980–2016	no	nd	NE	*Vulpes vulpes* (red fox)
FUR-15	1980–2016	yes	nd	E	No amplification
FUR-16	1980–2016	no	nd	E	*Neovison vison* (mink)
FUR-17	1930–1980	no	Siberian wolf	NE	*Canis latrans* *(coyote)*
FUR-18	1930–1980	no	Wolf	NE	*Canis latrans* *(coyote)*
FUR-19	1930-1980	no	Wild cat	NE	*Felis* spp. (domestic/wild cat)
FUR-20	1980–2016	no	nd	NE	*Lynx* spp. (lynx)
FUR-21	1980–2016	no	nd	NE	*Canis lupus* ssp. (dog/wolf)
FUR-22	1980–2016	yes	nd	NE	*Canis lupus* ssp. (dog/wolf)
FUR-23	1980–2016	yes	nd	NE	*Canis lupus* ssp. (dog/wolf)
FUR-24	1980–2016	no	nd	NE	*Alopex lagopus* (arctic fox)
FUR-25	1930–1980	yes	nd	E	No amplification

**Note:**

Abbreviations as in Table 1. nd, not declared; NE*, fur not classified. See text for details. Adapted from Table 3 in Garofalo et al., (2018).

**Figure 10:**
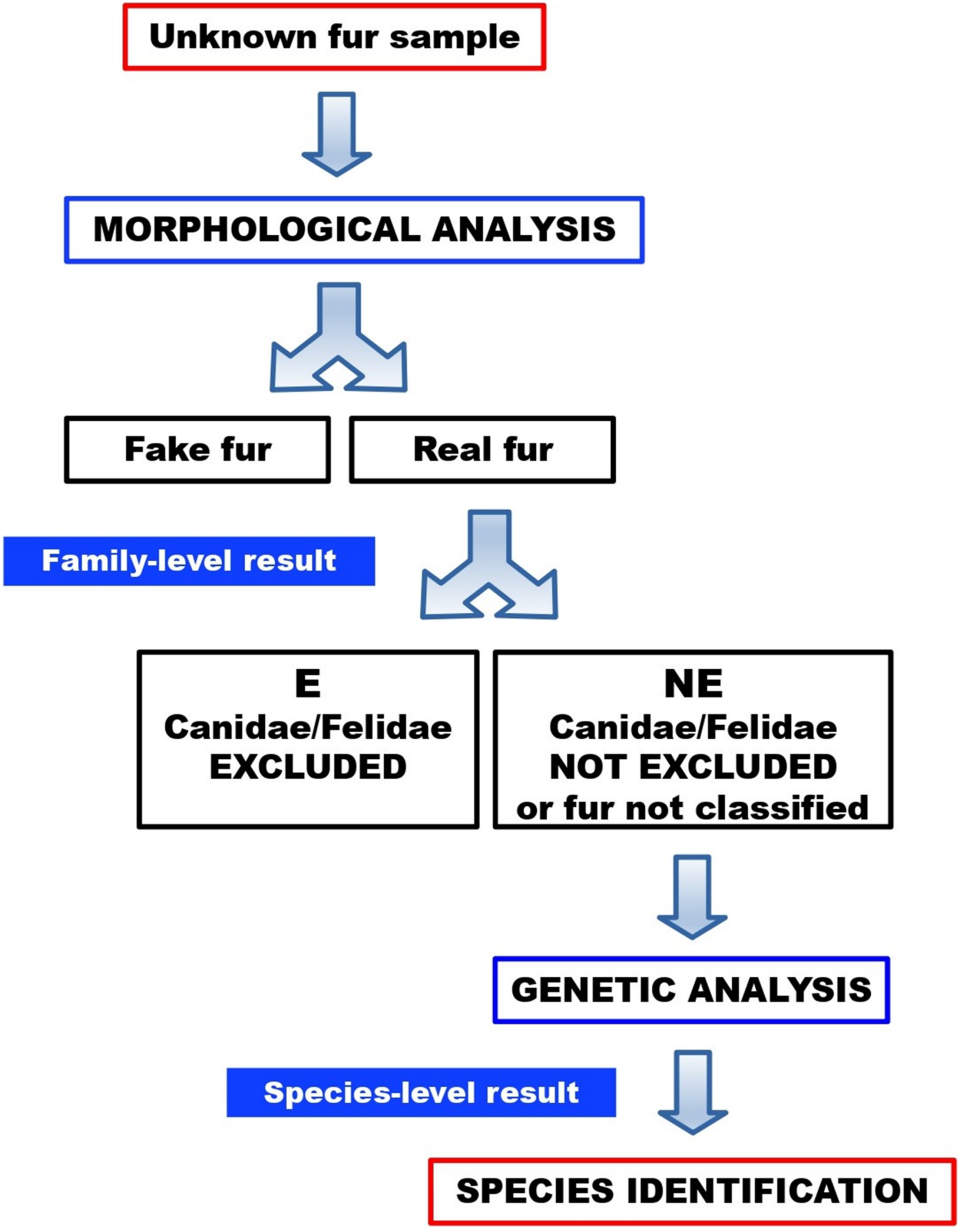
Workflow for the identification of illegal cat and dog furs, through our combined morphological and molecular approach.

No sample turned out to be a fake fur. Twenty-one furs (84%) were categorised as “not excluded”, therefore these samples could not be morphologically ruled out as dog or cat furs. One of these “not excluded” samples (FUR-12) could not be morphologically classified, due to an intense artificial dye that made it impossible to visualise and examine the medulla. Four furs (16%) were categorised as “excluded”, therefore these samples were morphologically ruled out as dog or cat furs. In a routine workflow (Fig. 10), the latter would not be submitted to molecular testing, since they were excluded as dog or cat furs. Here, however, these samples were molecularly tested to verify the reliability of morphological results.

Among the four “excluded” samples at the morphological screening, two were identified by the genetic analysis as belonging to *Sciurus* spp. (FUR-9) and *Neovison vison* (FUR-16) (Table 3). “Not excluded” samples from morphological screening were all confirmed by genetic analysis as belonging to species of Canidae or Felidae, including the one dyed sample that could not be fully examined at the morphological level. No conflicting results were observed, where both morphological and molecular results were obtained for casework samples.

## Discussion

EU Regulation 1523/2007 banned the trade of cat and dog furs (European Parliament, 2007), but no published reports are available on the actual effectiveness of the provided analytical methods or on their application in the following years.

Microscopic morphological identification of mammalian hairs poses several challenges due to marked somatic variation that occurs within species, breeds, individuals and among body regions in the same animal (Moore, 1988; Van den Broeck, Mortier & Simoens, 2000; Sahajpal et al., 2008; Davis, Brummer & Shivik, 2010; Czyz et al., 2012; Tridico et al., 2014). Identification at the species or even genus level requires extensive training and experience of the examiner (Ciucci & Reggioni, 2003; Tridico et al., 2014), application of multiple analytical methods (Teerink, 1991; Chernova, 2001; Chernova, 2003; Debelica & Thies, 2009) and a vouchered collection of reference specimens for comparison with questioned samples. Sometimes, even when all these conditions have been met, only an identification at the family level can be obtained with some degree of confidence (Gonzalez, 2011).

For all the above reasons, the use of morphological identification of animal hair as a single assay to identify dog and cat in furs to comply with the EU Regulation would be hardly feasible. Indeed, in order to test large numbers of commercial fur products on a national scale, a rapid and easily performed morphological screening test is needed. Since the microscopic examination of major structural hair features, such as the outermost cuticle and the central core (medulla), supports the exclusion of particular taxa as sources of a hair sample (Tridico et al., 2014), we chose to develop a simplified morphological criterion to assist in the exclusion of canid and felid species as sources of unknown fur samples.

The proposed morphological criterion is based on the microscopic observation of three main characteristics of guard hairs, namely (1) the cuticle pattern, (2) the medullar pattern (considering the medulla on all the length of the shaft) and (3) the medullar margins (considering the medulla at the thickest part of the shield region). Morphological analysis should be conducted on guard hairs, since these hairs are commonly acknowledged as the hairs that exhibit the highest number of diagnostic traits for microscopic identification.

Our morphological criterion can be applied to hairs of unknown body origin and morphologically altered by trimming or dying: this is an important feature of the proposed analysis, since hairs in commercial product can constitute very scarce samples, for example if used as decorations for small parts of clothing or soft-toys. This basic morphological analysis can be carried out by minimally trained personnel, allowing to put in place a surveillance network for a rapid and inexpensive exclusion of samples not consistent with dogs and cats, including fake furs. Such use of morphology for screening purposes disregards some of the steps that are otherwise mandatory when engaging in the morphological identification of animal hairs at the species level, such as the observation of macroscopic features (colour, banding, length, etc.), comparison with vouchered reference hairs coming from similar body regions, or distinction among different types of guard hairs (Pilli et al., 2014; Tridico et al., 2014).

Our morphological criterion was first tested on a collection of reference hair samples. The method proved to be effective overall, given that no incorrect exclusions of canids and felids occurred. Samples belonging to *P. lotor, M. meles* and *V. pacos* were incorrectly not excluded as dog/cat furs. These false positives are possibly due to different reasons. The medulla pattern of hairs from raccoon and canids can be similar, and therefore hardly distinguishable, especially if oils used for the sample mount during the setup of the slide do not penetrate into the medulla (Teerink, 1991). It was also reported that hairs of the European badger may lack the classical mustelids-like pattern, showing, for example, rather straight medullar margins in the thickest part of the shield, so that they closely resemble the hairs of felids (Teerink, 1991). Morphological traits from some of the horse, goat and alpaca samples that we analysed did not fit perfectly our selective criterion used to categorise hairs with certainty, possibly because of a rather amorphous medulla, and devoid of any diagnostic trait. Literature did not assist us in the explanation of these results, because published data on many species, especially the domestic forms, are lacking. However, for the purposes of this work, it is highly advisable that the morphological screening is such as to avoid type II errors (false negatives). In other words, from an enforcement perspective, it is desirable that the screening test is robust to false negative results, rather than a few false positives that can be identified at the molecular testing stage.

Our combined protocol was later tested on unknown evidence samples of furs/hides from real caseworks. DNA testing alone would have identified 84% of samples (with four failed amplifications out of 25 fur samples), while the combined protocol reached 92% classification (with only two failed amplifications of furs not previously excluded as canids or felids by morphology). Two furs, for which no DNA amplification was obtained, could already be excluded by morphological analysis as belonging to canid or felid species. In a routine workflow, 16% of samples (4/25 furs) would have therefore bypassed the DNA analysis thanks to the morphological screening. Eventually, molecular species identification of the 21 “not excluded” samples led to ascertain the presence of *C. lupus* ssp. in five samples, which were probably illicit furs (Garofalo et al., 2018).

While DNA analysis is highly effective in fur identification at the species level (Garofalo et al., 2018), molecular testing is also expensive and labor-intensive, so it would not be reasonable to apply it arbitrarily to all the casework samples. Furthermore, in samples where no DNA amplification can be obtained, it is possible to exclude canid or felid species with a previous cheaper morphological screening. Morphological preselection of animal hairs could, therefore, allow significant cost savings (Table 4), avoiding indiscriminate recourse to molecular analysis on all samples.

**Table 4:** Costs and running time of analytical methods. Methods used by EU Member States to identify the species of origin of fur, respective costs and running time (adapted from: European Commission, 2013).

Method	Costs (€)	Time
Microscopy	30–60	60−120 min
Species Identification of Animals (SIAM) by MALDI – TOF mass spectrometry	150–250	Few hours
DNA analysis	150–1075	7−10 days

## Conclusions

European Regulation 1523/2007 banned the trade of dog and cat furs, stating that different analytical methods (microscopy, DNA testing and mass spectrometry) can be used to exclude dogs and cats as source species, but an agreed analytical protocol was not provided. We explored the use of a combined morphological-molecular procedure, which represents a promising start to enforce the above Regulation. Our proposed microscopic morphological screening of hairs disregards some of the standard canons that would be mandatory in the morphological identification of animal hairs (in particular the macroscopic characteristics of the hair, and the comparison with a reference hair collection), as it does not aim at the precise identification of species but at the exclusion of canids and felids as sources of the questioned fur. Effectiveness of the two combined analytical methods proved to be overall higher compared to the single assays.

Morphological screening can be used for fast and inexpensive exclusion of synthetic furs and furs not belonging to canid or felid species, for an overall reduction of costs thanks to the lower number of samples to be submitted to DNA analysis. Infringements on the ban are punished with administrative and criminal sanctions (fines and imprisonment of different severity depending on EU Member State’s legislation). Criminal sanctions require that the case is referred to a court of justice, therefore an effective and validated analytical protocol should be necessary for the case to stand in court, to ensure justice and prevent wasteful appeals against the State in case of disputes.

## Additional Information and Declarations

### Data Availability

The following information was supplied regarding data availability: All data is available in the article and the tables.

## References

[ref-1] Alacs EA, Georges A, FitzSimmons NN, Robertson J (2010). DNA detective: a review of molecular approaches to wildlife forensics. Forensic Science, Medicine and Pathology.

[ref-2] Chernova OF (2001). Architectonics of the medulla of guard hair and its importance for identification of taxa. Doklady Biological Sciences.

[ref-3] Chernova OF (2003). Architectonic and diagnostic significance of hair cortex and medulla. Biology Bulletin of the Russian Academy of Sciences.

[ref-4] Ciucci P, Reggioni W (2003). Valutazione dell’affidabilità degli operatori per l’identificazione microscopica di peli di mammiferi. Hystrix: Italian Journal of Mammalogy.

[ref-5] Czyz K, Patkowska-Sokola B, Filistowicz A, Janczak M, Bodkowski R (2012). Analysis of hair coat of dachshund of longhaired, shorthaired, and wirehaired variety. Bulletin of the Veterinary Institute in Pulawy.

[ref-6] Davis AK, Brummer SP, Shivik J (2010). Sexual differences in hair morphology of coyote and white-tailed deer: males have thicker hair. Annales Zoologici Fennici.

[ref-7] De Marinis AM, Agnelli P (1993). Guide to the microscope analysis of Italian mammals hairs: insectivora, rodentia and lagomorpha. Bolletino di Zoologia.

[ref-8] De Marinis AM, Asprea A (2006). Hair identification key of wild and domestic ungulates from southern Europe. Wildlife Biology.

[ref-9] Debelica A, Thies ML (2009). Atlas and key to the hair of terrestrial Texas mammals.

[ref-10] European Commission (2013). Report from the Commission to the European Parliament and the Council on the application of Regulation (EC) No 1523/2007 banning the placing on the market and the import to, or export from, the Community of cat and dog fur. and products containing such fur. http://eur-lex.europa.eu/legal-content/EN/TXT/?uri=CELEX%3A52013DC0412.

[ref-11] European Parliament (2007). Regulation (EC) No 1523/2007 of the European Parliament and of the Council of 11 December 2007 banning the placing on the market and the import to, or export from, the Community of cat and dog fur, and products containing such fur. http://eur-lex.europa.eu/legal-content/en/ALL/?uri=CELEX%3A32007R1523.

[ref-12] Fediaf (2018). European facts and figures 2018. http://www.fediaf.org/images/FEDIAF_Facts__and_Figures_2018_ONLINE_final.pdf.

[ref-13] Garofalo L, Mariacher A, Fanelli R, Fico R, Lorenzini R (2018). Hindering the illegal trade in dog and cat furs through a DNA-based protocol for species identification. PeerJ.

[ref-14] Gonzalez MV (2011). HairbaseTM: the development of an online reference atlas of mammalian hair.

[ref-15] Gonzàlez-Esteban J, Villate I, Irizar I (2006). Differentiating hair samples of the European mink (*Mustela lutreola*), the American mink (*Mustela vison*) and the European polecat (*Mustela putorius*) using light microscopy. Journal of Zoology.

[ref-16] Hollemeyer K, Altmeyerb W, Heinzle E (2007). Identification of furs of domestic dog, raccoon dog, rabbit and domestic cat by hair analysis using MALDI-ToF mass spectrometry. Spectroscopy Europe.

[ref-17] Iyengar A (2014). Forensic DNA analysis for animal protection and biodiversity conservation: a review. Journal for Nature Conservation.

[ref-18] Johnson RN, Wilson-Wilde L, Linacre A (2014). Current and future directions of DNA in wildlife forensic science. Forensic Science International: Genetics.

[ref-19] Linacre A, Tobe SS (2011). An overview to the investigative approach to species testing in wildlife forensic science. Investigative Genetics.

[ref-20] Moore JE (1988). A key for the identification of animal hairs. Journal of the Forensic Science Society.

[ref-21] Moore TD, Spence LE, Dugnolle CE (1974). Identification of the dorsal guard hairs of some mammals of Wyoming.

[ref-22] People for the Ethical Treatment of Animals (PETA) (2019). The Chinese fur industry. https://www.peta.org/issues/animals-used-for-clothing/fur/chinese-fur-industry/.

[ref-23] Pilli E, Casamassima R, Vai S, Virgili A, Barni F, D’Errico G, Berti A, Lago G, Caramelli D (2014). Pet fur or fake fur? A forensic approach. Investigative Genetics.

[ref-24] Sahajpal V, Goyal SP, Jayapal R, Yoganand K, Thakar MK (2008). Hair characteristics of four Indian bear species. Science & Justice.

[ref-25] Teerink BJ (1991). Hair of west-European mammals. Atlas and identification key.

[ref-26] Toth M (2002). Identification of Hungarian mustelidae and other small carnivores using guard hair analysis. Acta Zoologica Academiae Scientiarum Hungaricae.

[ref-27] Tridico SR, Houck MM, Kirkbride KP, Smith ME, Yates BC (2014). Morphological identification of animal hairs: myths and misconceptions, possibilities and pitfalls. Forensic Science International.

[ref-28] Van den Broeck W, Mortier P, Simoens P (2000). Scanning electron microscopic study of different hair types in various breeds of rabbits. Folia Morphologica.

[ref-29] Wilson MR, Di Zinno JA, Polanskey D, Replogle J, Budowle B (1995). Validation of mitochondrial DNA sequencing for forensic casework analysis. International Journal of Legal Medicine.

